# The association between expressed emotion, illness severity and subjective burden of care in relatives of patients with schizophrenia. *Findings from an Italian population*

**DOI:** 10.1186/1471-244X-12-140

**Published:** 2012-09-13

**Authors:** Giuseppe Carrà, Carlo Lorenzo Cazzullo, Massimo Clerici

**Affiliations:** 1 Department of Mental Health Sciences, University College London, Charles Bell House. 67-73 Riding House Street., London, W1W 7EY, UK; 2 Association for Research on Schizophrenia (ARS), Via Andreani 4, 20122, Milan, Italy; 3 Department of Neurosciences and Biomedical Technologies, University of Milano Bicocca Medical School, Via Cadore, 48., 20052, Monza, Italy

**Keywords:** Schizophrenia, Family, Expressed emotion, Burden of illness

## Abstract

**Background:**

An appropriate understanding of the association between high-Expressed Emotion (EE) in family members of people with schizophrenia, patients’ and relatives’ correlates is needed to improve adaptation of psychoeducational interventions in diverse cultures. The aim of this study was to test the hypothesis that relatives designated as high EE would report higher subjective burden of care, and would be associated with objective variables that indicate greater illness severity i.e. number of previous hospitalizations and duration of illness.

**Methods:**

We performed secondary analyses of baseline data from a randomized controlled trial conducted in Italy.

**Results:**

High-EE relatives reported more subjective burden of care in disturbed behaviours and adverse effects areas, but did not perceive more deficits in social role performances. As regards illness severity characteristics, neither the number of previous hospital admissions nor the duration of illness was associated with high-EE. However, patients’ previous psychosocial functioning, as measured by educational attainments, seems to protect the relative from high-EE status.

**Conclusion:**

There is a need for cross-cultural comparisons of the subjective experience of distress and burden among high EE carers as a target for intervention, aimed at reducing family stress as much as improving patient outcomes.

## Background

The roles of families in the care of people with schizophrenia and the ensuing caregiver burden have been increasingly acknowledged in the research literature in the last three decades [1,2]. The construct of burden of care has two distinct components [3]. Objective burden of care is meant to indicate its effects on the household (such as effects on health, financial loss and daily chores), whereas subjective burden indicates the extent to which the caregivers perceive the burden of care. Cultural factors likely play an important role in determining both the perceived burden and relatives’ attitudes towards patients. Their contributions to subjective burden of care and ethnic-related issues have been studied in several contexts in the last few years [4]. Family members in the US white population are significantly more likely than African Americans to feel subjectively burdened by, and have rejecting attitudes towards, their relatives with schizophrenia, as well as to be less tolerant of loss of a productive contributory role. On the other hand, African Americans seem to be less tolerant of disruptive psychotic behaviours [5,6]. Furthermore, US Hispanic families seem to be more accepting of current disability [7], although with higher rates of depressive symptoms [8]. Similarly, evidence from Europe shows that the ways in which relatives cope with patients and the burden imposed by the caring role might be influenced by cultural factors which affect relatives' appraisal of the patients' situation, with major differences between Northern and Southern European countries [9-12].

On the other hand, the predictive ability of the family Expressed Emotion (EE) construct has been demonstrated in a variety of international community settings [13-15]. Research on EE in relatives of people with schizophrenia has indicated that such an index, as rated by the Camberwell Family Interview (CFI) [16], is predictive of relapse after hospital discharge [17]. However, although relatives’ perceptions of burden in caring for family members with schizophrenia are correlated with their high-EE attitudes [18-20], the available evidence highlights again the role of culture in the expression of critical or emotionally overinvolved attitudes [21]. These may be more culturally accepted in some ethnic groups [22], suggesting also the need for different threshold scores to define high or low EE in cross-cultural studies [23].

Finally, it may well be plausible that some of the illness characteristics of relapse-prone patients might prompt high levels of EE and subjective burden in family members, causing vulnerable patients to relapse [24]. It seems unclear which patient’s characteristics have the most impact on caregivers. Several studies found that positive psychotic symptoms are more burdensome [25-27], whereas others concluded that negative ones are more troublesome for the caregiver to deal with [28]. Possibly, greater illness severity as such may have a heavier impact on the caregiver’s perceived burden of care [26].

However, there are considerable differences in terms of family ties between Northern and Southern European countries. The latter are grouped together as “strong family ties countries” and contrasted with the “weak family ties countries” of Northern Europe and North America [29]. Following this cultural norm, the prolonged stay of children in their parents’ home is considered a distinctive character of a “strong” family, whereas Americans and West Europeans value individualism and independence between generations [30].

To the best of our knowledge, no study has explored in an Italian cultural context, behaviours, attitudes and relative’s burden regardless of the patient’s clinical patterns so far. A few distinctive features, such as the particularly strong association between high levels of EE and readmission rates, have been found in Italian samples [31]. Thus this specific culture might influence also EE attitudes and subjective burden. The relationships between high-EE, schizophrenic illness characteristics, and subjective burden in Southern European countries might well be different to those observed in Anglo-Saxon ones.

The present study was undertaken with a view to remedying these limitations and was designed to examine to what extent EE levels in relatives were related to their subjective burden of care imposed by the caring role, in a representative Southern European sample. The aim of this study was to test the hypothesis that, in this cultural context, relatives’ high EE would be associated with higher subjective burden of care, and also with objective variables that indicate greater illness severity, i.e. number of previous hospitalizations and duration of illness.

We performed secondary analyses of baseline data from a prospective, randomized trial which assessed the medium and long term outcomes of two programmes of family intervention for the care of schizophrenia, compared to standard community mental health care, in Italy [12].

## Methods

### Setting

The study was carried out in a non-profit, family advocacy and support agency, the *Association for Research on Schizophrenia* (ARS), which is supported by a charity (Cazzullo-Legrenzi Foundation) in Milan, Italy. The Lombardy Health System encourages non statutory charities, funded by the National Health Service (NHS), to complement existing teams by providing treatments that are not otherwise available. ARS provides programmes just for key-relatives of people with schizophrenia due to organizational and cultural barriers to patient’s participation. However, other relatives living with the patient in the same household are not offered the programmes. NHS community mental health centres in the metropolitan catchment area refer the relatives. A more detailed description of the different therapeutic options provided as well as details about sampling and randomization procedures are fully described elsewhere [12]. In brief, the different therapeutic options consisted of two elements. The first involved weekly meetings with an information group (IG) composed of 16–18 relatives for 24 sessions (1.75 h per session) using an informative approach. Curricula included: aetiology, positive symptoms, negative symptoms, mood disorders, problem behaviours, medical and psychiatric treatment, denial and non-compliance, interpersonal and social issues, relationship with family, education, independence and dependence, resources and benefits. Educational tools included lectures, videos and leaflets. The second element comprised weekly meetings for 48 sessions (1.5 h per session) over 2 years with a support group (SG), made up of 8–9 relatives who had previously attended the IG. This involved training on communication and coping skills, stress identification and management, and multiple family group-based problem solving, during the first year. In the second year mutual support was emphasized with deliberate efforts to mould the group into a social network that could persist for an extended period and satisfy family needs for social contact, emotional support, and ongoing monitoring through problem solving. Both programmes were co-led by two specifically trained psychiatrists not involved in patients’ community standard care.

### Participants

From those who had been referred to ARS consecutively from 1995 to 2000 (*n* = 320), relatives were selected with the following inclusion criteria (*n* = 205):

• they were living with someone suffering from schizophrenia and had not attended family groups or other support services before the study intervention;

• the patient was clinically stable (having had no psychiatric hospitalization or any relapse for six months prior to study entry) and was not receiving any psychosocial or rehabilitative treatment other than standard care;

• the patient did not have a primary diagnosis of alcohol or drug dependence or organic disease.

Relatives were randomly selected, using a random numbers table, to enter the study. In total, 101 out of 112 relatives agreed to participate, and gave informed consent. The family programs which were offered involved only one relative from each patient’s family, and all patients received standard care, which entailed key worker’s management and consistent pharmacological interventions monitored by consultant psychiatrists in community mental health centres of the Milan metropolitan area.

### Measures and procedures

Research assistants were not involved in the treatment and carried out the interviews at the office. Patient’s and caregiver’s data for this study were obtained at study entry before any intervention was given. Community-based service managers were contacted to check the following criteria: a) patients’ DSM-IV diagnoses of schizophrenia, as assessed by senior consultant psychiatrists [32]; b) current satisfactory functioning, as measured by a Global Assessment Scale-GAS score of 30 or more [33]; and c) consistency of prescribed pharmacological treatment, with all but 3 patients receiving standard doses (300–1000 mg chlorpromazine equivalents). In addition, at induction each relative was given a standardized questionnaire on clinical and social characteristics of the patient and family. Clinicians, including care coordinators and consultant psychiatrists, from the community settings supplied missing information on patients’ treatment variables if needed.

The relatives’ EE was evaluated by the Camberwell Family Interview-CFI [16]. Every interview was tape recorded. The two evaluators had been formally trained by Dr Christine Vaughn. Relatives were defined as high EE if they made six or more critical comments (CC), expressed hostility, or were rated as four or more on the Emotional Overinvolvment (EOI) scale in the course of the interview. The latter is in accordance with the Italian field study on predictive value [31] and not with the classical scoring criterion of 3 or more on EOI [34]. Positive remarks (a frequency count) and warmth (a 6-point scale: 0–5) were rated as well. The inter-rater reliability of the EE evaluators as regards the binary ordinal scale (high, low) was good (kappa = 0.86).

Subjective burden of illness over the previous 6 months was measured with the Social Behaviour Assessment Schedule (SBAS) [35,36]. The English language version of the SBAS scale was translated into Italian by native Italian speakers who are experts in psychiatric interviewing and/or psychiatric epidemiology following the official WHO forward-translation and back-translation protocol ( http://www.who.int/substance_abuse/research_tools/translation/en/). A native English speaker with a BSc in psychology from a UK university back-translated the Italian version into English. This back-translation was then checked for consistency of meaning with the original English version. This process was repeated until the back-translation was found to correspond to the original. Adjustments were also made to increase the clarity and precision of the Italian version of the questionnaire. Consensus about validity issues in the final version was reached with a focus group of experienced clinicians, though a formal validation procedure was not completed. SBAS is a validated, semistructured interview used to investigate the perceptions of caregivers regarding patient’s disorders and the caregivers’ subjective and objective burden. In terms of reliability, SBAS has shown intraclass correlation coefficients ranging from 0.92 to 0.99 [35] and weighted *κ*s between 0.83 and 0.98 [37] for the six subscales. In this study, only three of the six sections of the instrument were retained. The use of these sub-scales of the SBAS can be done without losing its psychometric properties [35]. The three sections and related dimensions dealt with: (a) disturbed behaviours; (b) change in social role performance; and (c) adverse effects of the illness on the household and the caregiver’s work and leisure time. The first section is concerned with eliciting a description of the patient's behaviour, including severity of disturbance, onset and distress caused to the informant. The second section has a similar scope with regard to the patient's social performance. The last section examines the consequences of the patient's behaviour and the subsequent emotional distress caused to the household. For each item, SBAS distinguishes between the objective change related to the occurrence of a problem, from the perceived distress, which is scored separately, and subjective burden caused. The level of distress reported by the relative, and created by each problem presented by the patient or existing within the household, ranges on a scale 0 = no distress, 1 = moderate distress, 2 = severe distress. Research assistants were trained in the use of the interview and coding, which were discussed in the group. Inter-observer reliability was evaluated using Cohen's kappa, with kappa values ranging from 0.82 for patient’s disturbed behaviours and 0.91 for change in social role performance. Different researchers conducted EE and SBAS interviews. The SBAS has 35 items from the 3 sections mentioned above, relevant to all informants, with 22 items on disturbed behaviours of patients, 5 items on social role performance and 8 items on adverse effects. We followed similar methodologies used in previous studies [38,39]. For each dimension, the mean distress score was computed as the sum of scores divided by the number of applicable items. The total score for subjective burden in each dimension ranged from 0.0 to 2.0.

### Ethics

The study was approved by the regional ethical review board in Milan, Italy and conducted according to the 1964 Declaration of Helsinki. All the participants signed an informed consent form.

### Statistical analysis

Analyses were carried out using STATA version 10 for Windows [40]. All statistical tests used the 5% level of significance, and all p-values were two-tailed. Descriptive analysis was followed by assessment of bivariate relationships between groups (low/high EE). T-tests were used for continuous variables. However the t-test for unequal variances was used, as the variances of the two subgroups examined were often not homogeneous when checked with one-way ANOVA, and the Welch’s approximation of the degrees of freedom was produced. Chi-square and Fisher’s exact tests were used for nominal variables. Secondly binary logistic regression with a stepwise procedure was used to analyze the association between the dichotomized outcome (low/high EE) and all the variables that were significantly related to caregiver’s high EE (p < .05) at the univariate analysis, together with hypothesized patient and family correlates as explanatory variables. The outcome variable was analyzed yielding odds ratios (ORs) with 95% confidence intervals (CIs), and p values. The goodness of fit for models was evaluated via Hosmer-Lemeshow test.

## Results

### Characteristics of patients and relatives

The overall mean age of patients was 29.8 years (SD = 8.6), and 28% were women. Furthermore, medium levels of education (mean years = 11.9; SD = 3.2) did not support consistent regular employment status (24/101) and only a few (11%) had stable intimate relationships, with most patients still living with their family of origin. The clinical profile corresponded to that usually reflected in studies of this type in terms of onset age (*M* = 20.2 years; SD = 6.7), duration of illness (*M* = 10.2 years; SD = 8.2), and number of previous hospitalizations (*M* = 3.3; SD = 5.1). Most of the key-relatives were parents (79%), middle-aged (*M* = 54.7 years; SD = 10.5), with similar medium levels of education (*M* = 9.8 years; SD = 3.9). They were generally mothers (71% overall), with noteworthy rate (77%) of high contact dichotomized as more or less than 35 hours per week. Thirty-nine relatives (39%) were rated high EE (24 women and 15 men). Within the high EE subgroup, critical relatives were mainly represented (81%), followed by hostile (68%) and EOI (53%) ones. Comparison of study participants and non-participants on all measures used in the study showed no significant differences.

### Relationship between EE levels and characteristics of relatives and patients

As regards the relationship between EE levels and characteristics of relatives and patients, there were few significant differences on all measures used in the study (Table 1). None of the socio-demographic characteristics of patients and relatives, except educational status of the patient, was statistically associated with EE level. Low-EE patients had spent significantly more years in formal educational programmes. The total mean score for high EE-relatives on distress as measured by SBAS was more than twice on disturbed behaviours section, *t* (88.67) = −5.35, but almost three times as high as the total mean score for low-EE relatives on social role performance, *t* (78.18) = −5.19, and adverse effects, *t* (86.99) = −6.50, dimensions, (*P* <0.0001 for all).

**Table 1:** Patients’ and relatives’ characteristics by level of Expressed Emotion

	**Low** **EE n = 62**	**High** **EE n = 39**	***P***
**PATIENTS**			
Age: Mean (SD), yrs.	30.4 (8.7)	28.8 (8.5)	NS
Gender: No. (%)			NS
Male	46 (74)	27 (69)	
Education: Mean (SD), yrs.	12.4 (3.4)	11.1 (2.7)	0.041^a^
Ordinary employed, No. (%)	17 (27)	7 (18)	NS
Married/cohabiting: No. (%)	7 (11)	4 (10)	NS
Living conditions: No. (%)			NS^b^
In parental home	51 (82)	29 (74)	
In conjugal home	6 (10)	3 (8)	
Alone	5 (8)	7 (18)	
Onset age: Mean (SD), yrs.	20.1 (6.3)	20.3 (7.3)	NS
Duration of illness: Mean (SD), yrs.	11.2 (8.6)	8.5 (7.2)	NS
Previous hospitalizations: Mean (SD), No.	3.2 (4.8)	3.4 (5.5)	NS
**RELATIVES**			
Relationship to Patient			NS
Parent	46 (74)	34 (87)	
Sibling	11 (18)	3 (8)	
Spouse/Partner	5 (8)	2 (5)	
Gender: No. (%)			NS
Male	20 (32)	15 (38)	
Age: Mean (SD), yrs.	54.4 (11.7)	55.1 (8.4)	NS
Education: Mean (SD), yrs.	9.87 (4.1)	9.84 (3.5)	NS
Relative’s hours per week spent in contact with the patient > 35: No. (%)	46 (74)	32 (82)	NS
SBAS distress scores: Mean (SD)			
Disturbed behaviour	0.66 (0.62)	1.30 (0.56)	<0.0001
Social role performance	0.37 (0.52)	0.94 (0.56)	<0.0001
Adverse effects	0.59 (0.55)	1.30 (0.52)	<0.0001

EE: Expressed Emotion; SBAS: Social Behaviour Assessment Schedule.

^**a**^t test with unequal variances, (Welch's degrees of freedom) = 2.0679, (95.2897).

^**b**^Fisher’s exact test.

### Multivariate analysis for the relationship between EE and explanatory variables

All significant correlates of high EE were entered into a stepwise multiple logistic regression model. Patient age and education were also investigated as possible confounders of caregiver burden. The first was included – given the high proportion of relatives among carers - because of its close association with the duration of relationship between carer and patient; the latter as a proxy measure of psychosocial functioning in terms of educational attainment in people with schizophrenia. Finally, as regards variables that indicate illness severity, patient’s number of previous hospitalizations and duration of illness were included.

Table 2 shows the model that best fitted the data, as the Hosmer-Lemeshow goodness-of-fit-test statistic was 7.3 (p > 0.50). Subjective burden scores were positively associated with high EE on disturbed behaviours and adverse effects dimensions, though on social role performance section scores did not reach the significance level. None of the clinical and socio-demographic variables of patients changed the associations between the above variables and high EE, apart from educational attainment - appearing to have some protective effect - with an odds ratio per year increase in formal education significantly lower than 1.0. Models using the most relevant EE components - EOI and CC dichotomized into high/low categories - as outcome variables, did not fit the data better than the overall EE measure.

**Table 2:** Variables associated with high EE in logistic regression

**Number of subjects included in the analysis**	**101**	
**LR**^**1**^	51.63	
**P**	<0.0001	
	**Odds ratio (95% CIs)**	***P***
**PATIENTS**		
Age	0.98 (0.90 to 1.07)	0.810
Education	0.80 (0.66 to 0.99)	0.040
Duration of illness	0.93 (0.84 to 1.02)	0.163
Previous hospitalizations	1.01 (0.90 to 1.14)	0.758
**RELATIVES**		
SBAS distress scores		
Disturbed behaviour	3.17 (1.11 to 9.07)	0.031
Social role performance	2.40 (0.77 to 7.42)	0.128
Adverse effects	4.79 (1.09 to 20.9)	0.037

^1^Likelihood Ratio.

## Discussion

### Main findings

The main findings of the present study are that high-EE relatives reported more subjective burden of care in disturbed behaviours and adverse effects areas, but did not perceive more deficits in social role performances. As regards illness severity characteristics, neither the number of previous hospital admissions nor the duration of illness remained associated with high EE in the regression analysis. However, patient’s previous psychosocial functioning, as measured by years successfully spent in formal education, seems protecting the relative from high-EE status. No other characteristics of relatives were associated with EE levels.

### Relationship between EE levels and clinical and socio demographic characteristics of patients

Our results are in agreement with other studies that examined the relationship between EE levels in relatives of people with schizophrenia and their characteristics at a single point in time. Several reports did not actually find any association between EE levels and demographic [18,41-43] or clinical [19,44-47] characteristics of patients. In our study, the educational status of the patient was the only demographic characteristic of patients and relatives which was statistically associated with, and found to be an independent predictor of, high EE. Although we found no univariate association between relative’s hours per week spent in contact with the patient, and EE status, we can presume that patients with higher educational attainments have had a larger social network, and less time to be actively engaged in the routine of the relatives. This in turn might either predispose or contribute to them being less critical of, or overinvolved with the patient. As a whole, once more, patients' functioning, rather than clinical characteristics, is a possible determinant of EE [48,49].

### EE levels and subjective burden of care

The study demonstrated in a realistically large Southern European sample that there is an association between relatives’ high EE and their subjective burden of care. This is consistent with most of [18], though not all [50], studies which used the SBAS, and different burden measures [43,51]. The two dimensions seem actually related and dependent on relatives’ appraisal of the patients’ condition rather than on his/her illness severity [18]. However, as measured by SBAS, only sections on disturbed behaviours and adverse effects of the illness on the caregivers’ work and leisure time remained statistically associated independent predictors of EE level, which was not the case for the social role performance area. The present results seemed to reflect this distinction in that there appeared to be a tolerance or resignation by relatives about social performance deficits, whereas patients’ (disturbed) behaviours and direct effects on relatives induced critical responses to a significant degree. In the context of our study, high EE in relatives of people with chronic illness seems more related to personal reactions to the direct and indirect tasks of care than to actual caregiving, which is the case for first episode psychoses [52]. If long-term carers believe that they are not in control of patient’s illness, they feel more stress and depression, have more negative views of the impact of care [53], and the lack of proactive strategies based on avoidant coping, may increase their levels of burden [54].

Although there appears to be broad agreement about the evidence that the EE-relapse association replicates, but is moderated, within different cultural contexts, there remains an increasing need to assess EE correlates and their significance internationally [24]. The prevalence of high-EE attitudes varies, with relatives of Indian and Latino patients being frequently classified as low rather than high [55,56] and levels of criticism significantly different across cultures [57]. Furthermore, ethnicity seems to influence the extent to which high criticism or EOI are culturally tolerable [22]. In particular EOI cannot be considered inevitably unfavourable as regards patients’ relapse risks, medicalizing what may be a cultural norm, though there is the need to balance the opposite risk it being ignored [58].

As much as the components of EE differ in relation to their predictive validity [24] and cultural significance [22], also the association between EE and burden may vary across different cultures. This study sought to examine such association in a non Anglo-Saxon cultural context. Key-relatives of people with schizophrenia in “strong family ties countries” [29] seem to be most burdened with patients’ disturbed behaviours and adverse effects of the illness on the caregiver’s work and leisure time. It seems important to understand cultural factors when planning and delivering interventions with the families of patients from distinct cultures [59]. EE should be regarded in an integrative model, in which the quality of the dyadic relationship, as assessed by EE, is the product of complex interactions between patients’ and relatives’ issues [19]. In our study the most burdensome issues seem related to the patient's disturbed behaviour and the adverse effects on the household, thus relevant family interventions need to focus on patients’ current, not past, characteristics. A problem-solving approach may show that the patient with psychosis is still capable of functioning as an adult. On the other hand, the more hostile and critical carers may positively react to information and advice, possibly on an ongoing basis within a group [60], by suggesting to them that patients’ thoughts and behaviours are not entirely under their control, being affected by symptoms of psychosis [61]. No improvement in relatives' burden may be realistically expected without specifically focusing on their appraisal of the patients’ condition regarding specific areas. Our study shows that in Southern European countries there is a need for interventions aimed at improving the impact of the caring role in areas of caregivers’ lives such as work and leisure time, as well as of behaviours which they perceive to be disturbing.

### Limitations and strengths of the study

The cross-sectional design of the study means that it is impossible to determine whether there is a causal relationship between EE and burden. Furthermore putting our results into the context of published research will be hindered by the variety of measures used about EE and family burden. However, we have used internationally validated instruments which would allow further sound replications [62], though patients’ outcomes were previously explored only in terms of clinical functioning and not in relation to level and severity of different symptoms [12].

The study was carried out at a non-profit agency in inner-city Milan, which is not part of statutory mental health services providing patient care, and this may limit its generalisability to other populations. Access was based on referral by community staff and such recruitment could have affected the generalisability of the findings. The relative’s motivation to accept family intervention not otherwise available could be similar to that in early family programs [63] and could have biased the results. Moreover subgroup analyses based on small numbers must be treated as preliminary. However, a relatively limited number of correlations were explored, so that the probability of chance findings was low, and more importantly the role of possible confounders has been addressed at the stage both of design (random sampling) and of analysis (use of multivariate statistical techniques and of goodness-of-fitness test to assess the models’ performance). Observer bias was unlikely, since different and mutually blind research assistants conducted EE and SBAS interviews.

## Conclusions

Despite general agreement about the effectiveness of family psychosocial interventions for the care of people with schizophrenia [64], there is the need to overcome organizational barriers whilst retaining basic components of successful family treatments for schizophrenia [65]. Consistently, further research should investigate the subjective experience of distress and burden among high EE carers as a target for intervention, reducing family stress as much as improving patient outcomes. Customs and traditions may define not only the sort of behaviours that warrant criticism [57], but also the burden linked to the relationship between patients and relatives as appraised by the latter, and every attempt should be made to pick up culturally sensitive issues [66,67] of maladjusted interactions between patients and carers [68]. Future cross-cultural comparisons might shed light on crucial adaptations in family psychosocial interventions.

## Competing interests

The authors declare that they have no competing interests.

## Authors’ contributions

GC planned the study, developed the measures, performed the data analysis and drafted the first version of the manuscript. CLC made substantial contributions to conception, acquisition of data, and commented on an earlier draft of the manuscript. MC made substantial contributions to conception, acquisition of data, and reviewed and revised the manuscript. All authors read and approved the final manuscript.

## Authors information

Carlo Lorenzo Cazzullo and Massimo Clerici to be considered as joint last authors.

## Pre-publication history

The pre-publication history for this paper can be accessed here:

http://www.biomedcentral.com/1471-244X/12/140/prepub
